# The ATP Synthase Deficiency in Human Diseases

**DOI:** 10.3390/life11040325

**Published:** 2021-04-08

**Authors:** Chiara Galber, Stefania Carissimi, Alessandra Baracca, Valentina Giorgio

**Affiliations:** 1 Consiglio Nazionale delle Ricerche, Institute of Neuroscience, I-35121 Padova, Italy; chiara.galber@phd.unipd.it (C.G.); stefania.carissimi@unipd.it (S.C.); 2 Department of Biomedical and Neuromotor Sciences, University of Bologna, I-40126 Bologna, Italy; alessandra.baracca@unibo.it

**Keywords:** ATP synthase, human disease, mitochondria

## Abstract

Human diseases range from gene-associated to gene-non-associated disorders, including age-related diseases, neurodegenerative, neuromuscular, cardiovascular, diabetic diseases, neurocognitive disorders and cancer. Mitochondria participate to the cascades of pathogenic events leading to the onset and progression of these diseases independently of their association to mutations of genes encoding mitochondrial protein. Under physiological conditions, the mitochondrial ATP synthase provides the most energy of the cell via the oxidative phosphorylation. Alterations of oxidative phosphorylation mainly affect the tissues characterized by a high-energy metabolism, such as nervous, cardiac and skeletal muscle tissues. In this review, we focus on human diseases caused by altered expressions of ATP synthase genes of both mitochondrial and nuclear origin. Moreover, we describe the contribution of ATP synthase to the pathophysiological mechanisms of other human diseases such as cardiovascular, neurodegenerative diseases or neurocognitive disorders.

## 1. Introduction

Mitochondria support aerobic respiration and produce the majority of cellular ATP by oxidative phosphorylation (OXPHOS) [1]. Electrons derived from the oxidation of fatty acids, carbohydrates and amino acids are shuttled to oxygen along the respiratory chain complexes (I–IV) embedded in the inner mitochondrial membrane (IMM), producing water and releasing the energy necessary to pump protons from the mitochondrial matrix to the intermembrane space (IMS). This results in the formation of a transmembrane electrochemical gradient across the IMM, which enables the ATP synthase to produce ATP from ADP and inorganic phosphate [2]. A reverse catalytic process can occur under anoxia, a condition in which ATP synthase couples ATP hydrolysis to the generation of a transmembrane potential [3,4]. The mitochondrial OXPHOS is the only metabolic pathway that is under dual genetic control. It is therefore possible to distinguish genetic defects caused by (i) alterations in mitochondrial DNA (mtDNA), ∼15%, e.g., Neuropathy, Ataxia, Retinitis Pigmentosa (NARP), Maternally Inherited Leigh’s Syndrome (MILS) and Leber’s Hereditary Optic Neuropathy (LHON) [5] and (ii) nuclear DNA (nDNA) mutations, which are inherited as Mendelian disorders. A recent review provided an update on the contribution of nuclear genes that impair mitochondrial respiration in patients and have been characterized in yeast [6]. More than 150 distinct genetic mitochondrial dysfunction syndromes characterized by a diminished OXPHOS capacity have been described [5,7,8,9,10,11]. Typical clinical traits include visual/hearing defects, encephalopathies, cardiomyopathies, myopathies, diabetes, liver and renal dysfunctions [12,13,14]. In other cases, mitochondria participate to the cascades of pathogenic events leading to the onset of several diseases, but they are not linked to their genetic origins. Mitochondria are damaged during the reperfusion of ischemic heart, age-related diseases and all the major neurodegenerative diseases––Parkinson’s (PD), Alzheimer’s (AD) and motor neuron diseases such as Amyotrophic Lateral Sclerosis (ALS).

**Figure 1:**
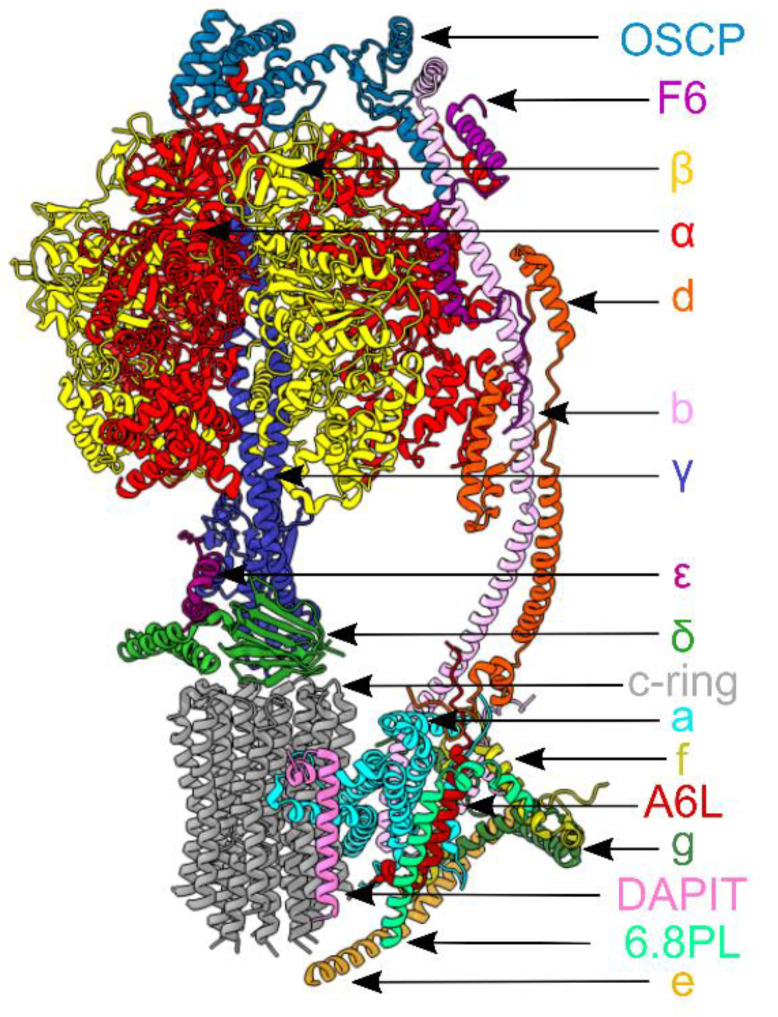
Subunit composition of the bovine ATP synthase monomer is mapped according to [15], (Protein Data Bank (PDB): 6ZQM). In the upper part, the subunits α(3) and β(3) of the catalytic domain are red and yellow, respectively; the three central stalk subunits γ, δ and ε, are blue, indigo and green. In the lower part, the membrane domain is composed of the c8-ring and the a subunit (dark grey and light blue); the supernumerary subunits e, f, g, A6L, 6.8PL and DAPIT are khaki, straw yellow, forest green, brick red, lime green and dark pink. On the right of the model, the peripheral stalk subunits OSCP, b, d and F6 are teal, light pink, orange and magenta.

In this scenario, ATP synthase has been shown to participate to the pathogenesis of different human diseases. The mitochondrial enzyme occupies the IMM of the organelle and forms dimers. Each monomeric unit (Figure 1), as shown in the latest dimeric mammalian enzyme by electron cryo-microscopy [15], is an assembly of 28 polypeptide chains of 17 different subunits organized into a catalytic globular domain, which is attached to an intrinsic membrane domain by a central stalk and a peripheral stalk [16]. This membrane-bound enzyme is a rotary machine. The membrane-bound rotor consists of eight identical c subunits (c- ring) in close association with a single a (or ATP6) subunit and is attached to the asymmetrical central stalk (subunits γ, δ and e) [17,18], which extends from the membrane domain and penetrates into the extrinsic globular catalytic domain along its central axis. As the central stalk rotates, it causes structural changes in the three catalytic sites, found mainly in each of the three β subunits, which alternate with three α subunits in the spherical extrinsic domain [16,19]. These structural changes lead to the enzyme’s catalytic activity. The peripheral stalk, composed of the subunits oligomycin sensitivity conferral protein (OSCP), b, d, F6 and the membrane extrinsic region of A6L (or ATP8), links the external surface of the catalytic domain (F_1_) to the a subunit in the membrane domain (F_o_) [20,21]. The subunits e, f, g, A6L and 6.8 proteolipid also contribute to the membrane domain of the peripheral stalk [22,23,24], and in the dimeric complex, some of them are involved in forming the interface between monomers [24]. Another subunit, previously known as diabetes-associated protein in insulin sensitive-tissues (DAPIT) [23], may be involved in the formation of links between dimer units in the rows of dimers [25]. In this review, we mainly focus on mutations in mitochondrial and nuclear genes encoding ATP synthase subunits and factors important for their association to human diseases. Moreover, we describe the contribution of this enzyme to the pathogenic mechanisms of cardiovascular, neurodegenerative diseases and neurodevelopmental disorders. Due to space constraints, the modulation and regulation of the ATP synthase in cancer has not been addressed in this review; however, for details, see [26,27].

## 2. Gene Mutations of ATP Synthase and Its Assembly Factors in Human Disease

Disorders caused by ATP synthase deficiencies can be classified depending on the mitochondrial or nuclear genetic origin (Table 1). These diseases are often severe encephalo- or cardiomyopathies and manifest shortly after birth. Interestingly, they are less frequent than other OXPHOS-related diseases [28].

### 2.1. Mitochondrial Gene Mutations of ATP Synthase

The better characterized ATP synthase diseases are caused by mutations in the mtDNA *ATP6* and *ATP8* genes [28], encoding for the human a and A6L subunits, respectively. The open reading frame of these two subunits overlap for 46 nucleotides; thus, changes occurring in this region can affect the expression of both subunits [44,45,46,47,48]. The majority mutations that cause defects of ATP synthase involve the *ATP6* gene. We focused our attention here on ATP synthase mutations, at the level of the *ATP6* gene, leading to altered enzyme functions that have been characterized, while we refer to other detailed reviews [11,28,60,61] and www.mitomap.org (accessed on 7 April 2021) for updated lists for the other *ATP6* mutations.

The most common of these mutations are the m.8993T>G/C (p.Leu156Arg/Pro) and the m.9176T>G/C (p.Leu217Arg/Pro) substitutions, which cause different clinical phenotypes varying from NARP to MILS, depending on mtDNA heteroplasmy [28]. These four mutations compromise mitochondrial ATP production with different degrees of severity and have also been modeled in yeast, in order to better clarify their role in the ATP synthase activity and assembly [34,35,62,63]. Not surprisingly, mutations in the yeast mitochondrial *ATP6* gene impair the ATP synthase function, since the a subunit is involved in the formation of the proton channel at the interface with the c-ring, which is formed in the F_o_ sector of the enzyme and is fundamental for the catalytic activity [15,64]. Another common mutation is the m.9035T>C, reported in studies of large patient cohorts [36,65,66]. Although a high level of heteroplasmy in patients has been shown to be required to develop a phenotype [36,66], the m.9035T>C mutation causes lower ATP levels, decreased ATP hydrolysis and increased reactive oxygen species (ROS) in patient tissues [42].

As clearly reported, the mtDNA 8993T>G mutation is associated with a more severe NARP/MILS clinical phenotype than the 8993T>C mutation [28]. Biochemical studies aimed at elucidating the pathogenic mechanisms of the two mutations showed that, in NARP/MILS patient cells harboring a high mutant load (>80%), the ATP synthase activity was drastically reduced (about 70%) and only slightly affected (about 20%) compared to the controls, when the mutations were the 8993T>G and 8993T>C, respectively [29,30,31]. Although, both mutations lead to cellular energy deficiency and increased ROS levels, the latter was reported as a major contributor to the pathogenesis of the NARP/MILS associated to the 8993T>C mutation [30]. In addition, a high percentage of 8993T>G mutation did not significantly affect either the ATP hydrolytic activity or the ATP-driven proton transport in mitochondria of patient cells, excluding that the mutation affects the assembly of the ATP synthase complex [32,33]. However, biochemical analyses in NARP/MILS lymphocytes revealed that the Leu156Arg mutant a subunit slightly affected the proton translocation through the enzyme, suggesting that the coupling between proton translocation through F_o_ and ATP synthesis on F_1_ was altered in the mutant ATP synthase complex [29]. These studies also suggested a close relationship of biochemical defect and tissue heteroplasmy. In addition, the clinical phenotype associated to mutations at both 8993 and 9176 nucleotides was found to be worsened by defects in respiratory complex function and assembly [67]. These findings elucidate plausible factors that might contribute to the difference in severity of the clinical phenotype associated with MILS and NARP, which the alteration in ATP synthase alone was unable to explain. The rescue of the energy deficiency that characterizes the cells of NARP and MILS patients has been positively targeted by both genetic and biochemical approaches providing different tools for the development of therapeutic strategies for patient treatment [68,69,70].

The m.9185T>C or m.9191T>C mutations of *ATP6* are variants of the early described NARP-MILS clinical spectrum. In both cases, leucine is changed into proline, at position 220 or 222 in humans, respectively, near the C-terminus of the protein. The first mutation was reported in many patients with a mild clinical phenotype [37,38] and was associated with decreased Mg^2+^-ATPase activity in isolated muscle mitochondria but normal respiratory chain enzyme activity [37,38]. The second mutation was instead discovered in a two-year-old patient who died presenting a severe clinical phenotype. This second mutation caused a severe reduction in Mg^2+^-ATPase activity accompanied by a decrease in the mitochondrial respiration rate, indicating a possible reduction also in ATP synthesis [53].

The *Saccharomyces cerevisiae* yeast equivalent of the m.9185T>C mutation (p.Ser250Pro, corresponding to human p.Leu220Pro) partially impaired the yeast ATP synthase activity with a 30% decrease in mitochondrial ATP production without any evidence of a proton leak [39]. The equivalent of the human m.9191T>C mutation (p.Leu252Pro in *S. cerevisiae*) instead caused a more severe dysfunction in terms of a >95% decrease in the ATP synthesis rate accompanied by a defective ATP synthase assembly. Subcomplexes of the ATP synthase and free F_1_ were detected by BN-PAGE analysis, suggesting that the mutant a subunit was not stably incorporated in the enzyme complex and, therefore, degraded. Since the proline amino acid is indeed a well-known α-helix breaker residue, a possible explanation of the described impaired ATP synthase assembly might reside in the fact that this mutation prevents the correctly folded structure of the a subunit and alters its proper interaction with the c-ring [39]. 

Another less frequent mutation is the *ATP6* m.8969G>A transition, which leads to the replacement of a highly conserved serine residue at position 148 of the human sequence with asparagine. This mutation was found in a six-year old male with Mitochondrial Myopathy, Lactic Acidosis and Sideroblastic Anemia (MLASA) [40], and in a 14-year old female with a severe nephropathy, carrying a high mutation level (>89%) in the kidney [57]. Biochemical investigations of mutant yeast and human cells revealed a decreased basal and oligomycin-sensitive respiration [40,41], indicating that the substitution of this serine into an asparagine severely compromised the ATP synthase activity, with a block of the proton transfer through the F_o_ [41]. Later on, it was shown that these detrimental consequences are caused by the amino acid substitution for asparagine. According to the authors, the asparagine (Asn175 in yeast), which replaces the serine of the normal sequence, binds (with the hydrogen bond) and neutralizes the nearby glutamate (Glu172 in yeast), which is critical for the proton flux in yeast ATP synthase [42]. 

A novel frameshift mutation (m.8611_8612insC) in the *ATP6* gene was discovered in 2017 in a patient with ataxia and encephalopathy symptoms [43]. A biochemical analysis revealed impaired assembly and accumulation of subcomplexes of ATP synthase, decrease in the enzyme activity and altered mitochondrial ultrastructure with aberrant cristae formation. All these features were attributed to an aberrant a subunit translation, with the consequent formation of a truncated form [43]. 

Mutations in the *ATP8* gene are less common. The mutations that cause ATP synthase deficiency are those occurring in the overlapping region of the *ATP6* and *ATP8* genes, thus interfering with the synthesis of both subunits. Two different nucleotide substitutions, m.8528T>C and m.8529G>A, both affecting the first amino acid residue in the human a subunit, and the amino acid residue in position 55 of the human A6L subunit (m.8528T>C (a subunit p.Met1Thr and A6L subunit p.Trp55Arg), m.8529G>A (a subunit p.Met1Ile and A6L subunit p.Trp55*)), were identified in patients suffering from severe cardiomyopathies [44,45,46]. The other two mutations, occurring in the overlapping region in the nucleotide m.8561 caused amino acid residue changes in position 12 and 66 of the human a and A6L subunits, respectively (m.8561C>G (a subunit p.Pro12Arg and A6L subunit p.Pro66Ala) and m.8561C>T (a subunit p.Pro12Arg and A6L subunit p.Pro66Ser)). These mutations were detected in individuals who had also early-onset ataxia and severe neurological signs [47,48]. In all these reported cases, the ATP synthase deficiency was due to an altered enzyme assembly that causes a consequent increase in the F_1_ subcomplex [46,47,48] and a decrease in ATP production [44,45,46,47,48]. 

### 2.2. Nuclear Gene Mutations of ATP Synthase and Its Assembly Factors

Mutations in the nDNA genes encoding for ATP synthase subunits are very rare. Only a few different cases have been discovered over the years. The first mutation was reported in the *ATP5F1E* gene, which encodes ε subunit in the central stalk of the enzyme [49]. More recently, different mutations were found in the *ATP5F1A (*α subunit) [50,51], in the *ATP5F1D* (δ subunit) [63] or in the *ATP5MK* (DAPIT subunit) [64] genes. These nDNA mutations cause a similar and marked decrease in the content of fully assembled ATP synthase complexes, with a consequent decrease in their activity [49,50,51,52] and are detailed below. 

The first case, the mutation causing Tyr12Cys amino acid residue substitution in the ε subunit, was described in a 22-year old woman presenting a neonatal-onset lactic acidosis, 3-methylglutaconic aciduria, mild mental retardation and developed peripheral neuropathy [49]. An analysis on patient fibroblasts with the homozygous missense mutation c.35A>G showed a decrease in the mitochondrial ATP synthase activity (both in ATP synthesis and hydrolysis) and assembly, caused by a reduction in the enzyme subunit level. Unlike the expression of the other subunits, the c subunit was found to accumulate and aggregate in a detergent-insoluble form, an accumulation that was also described in other disorders, such as Batten disease and fragile X syndrome, which will be discussed below. Overall, these findings suggest that the ε subunit is important for proper biosynthesis and assembly of the ATP synthase and for the proper incorporation of the c subunit into the rotor structure [49]. In line with these results, the downregulation of the ε subunit in HEK293 cells [71] or in yeast [72], caused a decrease in mitochondrial content and the activity of ATP synthase, with an effect on c subunit accumulation in HEK293 cells depleted of the ε subunit [71]. 

One of the other mutations (c.985C>T) of the nuclear *ATP5F1A* gene encoding the α subunit mentioned above was found in two different siblings who died in the first weeks of life [50]. The severity of this phenotype depends on the fact that the wild-type allele of the mother was not expressed in the siblings. Patient fibroblasts showed a reduction in the oxygen consumption rate, possibly caused by impairment of ATP synthase assembly and function. These cells displayed, in line with a decreased enzyme assembly, a decreased content of the subunits α, β, OSCP or d, which are important for the enzyme catalysis [50]. The authors showed that the expression of the wild-type gene encoding the α subunit in patient fibroblasts rescued the ATP synthase complex content and activity [50]. The possible explanation of the functional effects of the described mutation proposed by the authors implies that the substitution of arginine 329 for cysteine abolishes the three α-β interactions in the catalytic core of the enzyme, with the consequent loss of stability of the entire ATP synthase complex [50]. Another case is the homozygous c.962A>G mutation in the *ATP5F1A* gene that was described in two sisters born from consanguineous first-cousin parents. They both died early after birth with microcephaly, pulmonary hypertension and heart failure [51]. Patient muscle tissue showed OXPHOS deficiency and mtDNA depletion. Additionally, in this case, the p.Tyr321Cys mutation involved a highly conserved residue. The expression of the analogous yeast variant (*ATP1*: p.Tyr315Cys) in an *ATP1* knockout strain reflected the same severe phenotype with mtDNA loss, decrease of mitochondrial membrane potential and petite phenotype [51] 

Other examples of nuclear mutations known to cause a mitochondrial dysfunction involved the δ subunit and its *ATP5F1D* gene. Two different homozygous mutations, c.245C>T (p.Pro82Leu) and c.317T>G (p.Val106Gly), were found in two unrelated individuals with a metabolic disorder [52]. Cultured skin fibroblasts from these individuals showed an impaired ATP synthase assembly, as revealed through BN-PAGE, and decreased enzyme activity. Moreover, in both subjects, the amount of the δ subunit was unchanged but not that of other subunits like α, β or OSCP, which were decreased in abundance. Through in silico modeling, the authors found that each of the amino acid substitutions induces changes in the predicted structure of the protein. According to these data, they hypothesized that these changes can alter the ability of the δ subunit to bind and interact with the F_1_ subunits and thus affect the proper assembly of the enzyme. The pathogenicity of the two *ATP5F1D* variants was corroborated by studies performed in Drosophila. Indeed, both the mutated proteins were unable to complement the phenotypic defects caused by the δ subunit depletion in Drosophila, whereas the human wild-type subunit did [52]. Interestingly, and in line with the effect on the enzyme assembly, fibroblasts from the patient with a c.245C>T mutation showed significant decrease in mitochondrial cristae content [63], a fact consistent with the role of ATP synthase dimers in maintaining normal mitochondrial cristae morphology [73,74].

Recently, a novel homozygous splice-site mutation (c.87+1G>C) in the ATP synthase *ATP5MK* gene (encoding the DAPIT subunit) was described in three unrelated Ashkenazi Jewish families. The mutation negatively affects enzyme dimerization and ATP synthesis rate. Rescue with wild-type *ATP5MK* cDNA in patient fibroblasts restored the DAPIT protein levels, and enhanced ATP synthase dimers and their activity [53].

The biosynthesis of the eukaryotic ATP synthase is a highly organized process that requires the action of specific assembly factors [56,75,76,77,78,79]. It was shown that mutations in some of these “chaperone” proteins, named ATPA12 and transmembrane protein 70 (TMEM70), can be responsible for secondary ATP synthase deficiencies [80], leading to altered assembly and compromised activity of the enzyme.

The ATP12 protein is known to interact with the unassembled α subunit and is essential for its incorporation into the ATP synthase complex [78]. In a genetic study, De Meirleir et al. discovered a homozygous T>A missense mutation in exon 3 of the *ATPAF2* gene in a girl [54]. This mutation caused the amino acid substitution of a conserved tryptophan to an arginine at position 94 (p.Trp94Arg), which decreased the solubility of the protein with a tendency to aggregate [55]. The consequence is a severe decrease in the ATP synthase complex assembly and activity [54], even if no alteration in the mitochondrial morphology was observed in the fibroblasts derived from the patient carrying this mutation [55]. 

The other important regulatory protein in ATP synthase assembly is TMEM70, localized in the inner mitochondrial membrane [77]. Different mutations have been found over the years for *TMEM70*, with a broad spectrum of phenotypes and severity. The most common features of the syndrome caused by *TMEM70* mutations are a severe neonatal lactic acidosis, 3-methlyglutaconinc aciduria, cardiomyopathy, facial dysmorphism and mental retardation [7,81]. Early evidence for the role of TMEM70 in the enzyme assembly came in 2008 [56] and was later confirmed when it was shown that TMEM70 promotes the ATP synthase assembly by interacting with subunit c. This interaction facilitates the incorporation of the c subunit into the rotor structure of the enzyme within the inner mitochondrial membrane [76,77]. The c.317–2A>G mutation, which was firstly reported at the end of the second intron of the *TMEM70* gene, resulted in aberrant splicing and the loss of the transcript. As a consequence, low ATP synthase activity and assembly were observed. Fibroblast carrying this mutation were complemented with the wild-type *TMEM70*, which rescued structural and functional changes of ATP synthase, suggesting, for the first time, the importance of TMEM70 in the enzyme assembly [56]. All patients affected by this common mutation that were later diagnosed and exhibited ATP synthase deficiencies similar to the aforementioned case [57,58,59]. Other, less-common mutations on the *TMEM70* gene were found in many patients from different ethnic groups, with various phenotypes ranging from the absence of TMEM70 protein due to the premature stop codon, to the synthesis of an incomplete truncated form of the factor, lacking functional or structural domains [7,58,81,82,83,84]. As expected, a mitochondrial defect characterized by a decrease in ATP synthase assembly and activity was described in these patients [58,83,84]. Mitochondrial ultrastructural analysis in some *TMEM70* mutant samples showed a fragmented mitochondrial network and impaired mitochondrial morphology, swollen mitochondria or altered and concentric cristae [57,58,59,84] in line with the role of properly assembled ATP synthase in cristae shaping [73,74]. However, it has been shown that the disrupted mitochondrial cristae architecture in some patients also impairs the activity and localization of other OXPHOS complexes, increasing the severity of the disease [57,58,84]. Importantly, the mitochondrial effects caused by *TMEM70* mutations could be completely restored by complementation with the wild-type gene [84]. 

## 3. ATP Synthase Dysfunctions in other Human Diseases

**Figure 2:**
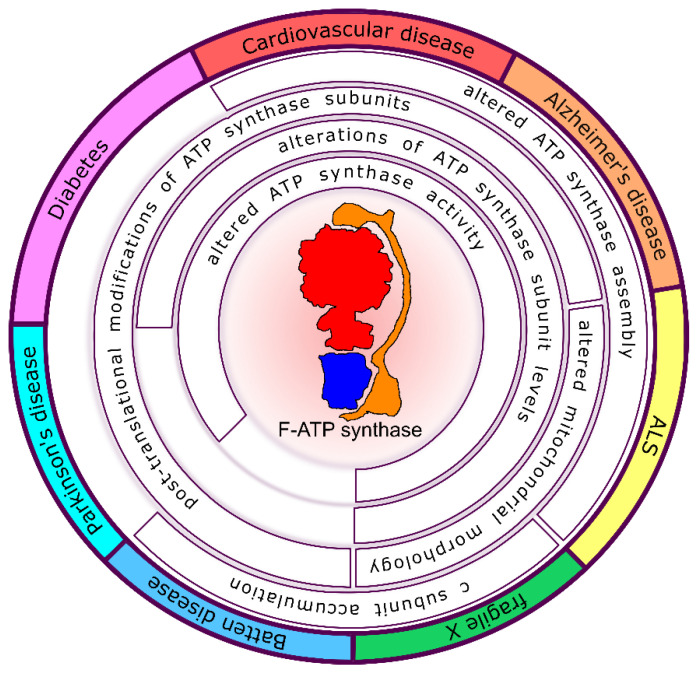
Schematic representation of the ATP synthase modifications involved in the progression of human diseases. Cardiovascular, Alzheimer’s, Amyotrophic Lateral Sclerosis (ALS), fragile X, Batten and Parkinson’s diseases are shown with different colors in the external perimeter. Changes in ATP synthase catalytic activity, assembly, subunit levels or subunit post-translational modifications and their consequence on mitochondrial morphology are listed inside the circle in correspondence of each related disease.

The ATP synthase dysfunctions involved in the pathogenic events, leading to cardiovascular, neurodegenerative and neurocognitive diseases are described below and shown in Figure 2.

### 3.1. Cardiovascular Disease and Cardio-Protection

Myocardial cell death due to ischemia–reperfusion is a major cause of morbidity and mortality in western nations. Rouslin has first demonstrated downregulation of the mitochondrial ATP synthase activity in ischemic heart tissue from different animals [85,86]. Under ischemic condition, the ATP synthase works in reverse hydrolyzing ATP. Thus, inhibition of the ATP synthase hydrolytic activity under these conditions conserves cellular ATP levels. The membrane potential prevents uncontrolled influx of ATP into the mitochondrial matrix via the electrogenic ATP/ADP translocator, thus limiting ATP hydrolysis. Furthermore, it is stated that during ischemia, the mitochondrial ATPase inhibitor protein (IF1) binds to and inhibits the mitochondrial ATPase, thereby conserving ATP [85,87,88,89]. IF1 can also contribute to the myocardial ischemic preconditioning, reducing the mitochondria damage during early reperfusion [90]. Cyclophilin D (CyPD), the permeability transition pore modulator, which also inhibits the ATPase catalytic activity [91], may contribute in preventing ATP dissipation as an additional mechanism of ATP synthase modulation. 

The reintroduction of oxygen during reperfusion allows the generation of ATP, but the damage to the electron transport chain results in increased mitochondrial generation of ROS. The catalytic activity of ATP synthase has been shown to be regulated in the presence of ROS in several cardiovascular studies, a fact which might be caused by oxidation of specific enzyme residues [92]. Mitochondrial Ca^2+^ overload and increased ROS can result in opening of the mitochondrial permeability transition pore, which further compromises cellular energetics and induces cell death. Apart from permeability transition-directed therapies [93], the cardioprotective strategy of ischemic preconditioning (PC), first described in 1986, provided an indication of the magnitude of the possible cardioprotective effect [94]. The rate of ATP consumption during ischemia is slower in PC hearts [95,96]. The full mechanism by which PC reduces ATP break down during ischemia remains still unknown. Interestingly, cardiac-specific overexpression of the antiapoptotic protein Bcl-2, overexpression of the cardioprotective PKC-ε and adenosine pretreatment have all been shown to slow the rate of ATP breakdown during ischemia [97,98,99]. Di Lisa et al. used the fluorescent membrane potential-sensitive dye JC1 to measure mitochondrial Δψ in anoxic rat cardiomyocytes and showed a biphasic decline in Δψ [100]. These authors showed that glycolytically generated ATP was used to maintain Δψ, since Δψ was shown to decline more rapidly during ischemia in the presence of oligomycin, an ATP synthase inhibitor. Leyssens et al. obtained similar results using JC1 to measure Δψ in rat cardiomyocytes metabolically inhibited with cyanide and 2-deoxyglucose [101]. These data support the conclusion that ATP synthase is a major consumer of ATP during ischemia and/or metabolic inhibition, and they further demonstrated that the consumption of glycolytic ATP is used to maintain Δψ [87]. 

It was proposed that PC promoted earlier binding of the IF1 to ATP synthase. However, studies by two different groups using submitochondrial particles found no evidence supporting inhibition of the ATPase in PC hearts [102,103]. Other groups, on the other hand, have reported that PC and diazoxide enhance the IF1 binding to ATP synthase [104,105,106]. A previous study has also reported that pharmacological PC with adenosine results in increased phosphorylation of the β subunit of the ATP synthase [107], although the functional effects of phosphorylation on the ATPase activity were not addressed. Subsequent studies aimed at generating different yeast mutants to better define the role of the β subunit phosphorylation, demonstrated its impact on enzyme assembly/stability and catalytic activity [108].

Additionally, changes in the amount of ATP synthase subunits have been shown in other cardiac patients. During an inflammatory cardiomyopathy occurring in patients affected by chronic Chagas disease, the most characteristic histopathological lesions are consistent with inflammation and a myocardial remodeling process such as T-cell/macrophage-rich myocarditis, hypertrophy and fibrosis [109]. Protein analysis showed a 20% decrease in the ATP synthase subunits α and β in the myocardium of chronic Chagas patients compared to myocardial samples from individuals without cardiomyopathies [110]. Since the analysis of the mRNA levels did not show significant differences [110], it seems plausible that a modulation at the level of subunit turnover or enzyme assembly might occur in chronic Chagas patients.

### 3.2. Neurodegenerative Diseases

The neurodegenerative diseases include AD, PD, ALS and Multiple Sclerosis (MS), injury to the central nervous system (CNS) through chronic low-grade hypoxia, the rarer Huntington’s disease (HD), Wilson’s disease and Freidreich’s Ataxia. In all these diseases, impaired ATP generation causes a failure of cellular homeostasis, with a number of consequences, including the ionic imbalance, altered Ca^2+^-dependent transmission of information in the CNS and ultimately, necrotic or apoptotic cell death, depending on ATP depletion. 

The first study to implicate ATP synthase in AD etiology found decreased level of the entire complex in the hippocampal tissue of AD patients through BN-PAGE analysis [111]. Whether the impaired complex stability in AD patients during detergent extraction also reflects changes in the catalytic activity of ATP synthase is still debated in the literature. An early study found no significant decrease in the ATP synthase catalytic activity in isolated mitochondria from AD patient hippocampal tissue, motor cortex and platelets [112]. However, there is evidence of post-translational modifications occurring in AD with consequences on the ATP synthase activity. In the hippocampus of AD patients, the α subunit was shown to be excessively nitrated in comparison to age-matched control brains [113], a post-translational modification that has been shown to inhibit the ATP synthase catalysis [92]. The α subunit can be subjected to glycosylation with O-linked β-N-acetylglucosamine (O-GlcNAcylated) on the Thr432 residue. This modification was reduced in brains of AD patients, Tg AD mice and in Aβ-treated mammalian cell cultures–resulting in reduced ATP levels [114]. Molecular modelling and co-IP experiments with deletion mutants of the α and β subunits showed that Aβ directly blocks the O-GlcNAcylation of this Thr432 residue. Interestingly, the O-GlcNAcylation of Thr432 that had been inhibited by Aβ was rescued by treatment with the O-GlcNAcase inhibitor. These findings are particularly noteworthy as the authors demonstrated a chemical mechanism for the interaction of the Aβ peptide with mitochondrial ATP synthase, which could provide a potential therapeutic target for AD [114]. Another post-translational modification was shown in the presence of a lipid peroxidation product, 4-hydroxy-2-nonenal (4-HNE) [115]. This product was shown to modify the α subunit of ATP synthase. It caused a 35%-decrease of ATP hydrolysis in the hippocampal tissue of early stage AD individuals with mild cognitive impairment [116] and a 30%-decreased ATP synthase activity in the entorhinal cortex [117], suggesting that oxidative stress precedes the presence of Aβ in the affected tissue. 

Multiple studies have pointed to a decrease in the levels of ATP synthase subunits in AD models. A decreased expression was observed in several of the nuclear ATP synthase genes in the posterior cingulate cortex, hippocampal field CA1, middle temporal gyrus, entorhinal cortex and posterior cingulate neurons [118]. A study using induced pluripotent stem cell (iPSC) -derived hippocampal neuronal cells, with familial associated presenilin 1 (PS1) mutation M146, observed a decreased level of the ATP synthase complex while PS1 expression was kept at physiological levels [119]. Neuroblastoma cells expressing the *ApoE4* allele of the *ApoE* gene, the major genetic risk factor for sporadic AD, showed a reduction in the levels of all detected ATP synthase subunits [120]. A reduced expression of the catalytic β subunit mRNA levels by over 50% was found in the mid-temporal cortex of AD patient brains [121]. In another study linking Aβ peptides with ATP synthase in AD, rats receiving a bilateral intrahippocampal injection of Aβ showed a significant decrease in the levels of β subunit of ATP synthase [122]. Gene expression analysis of the entorhinal cortex of AD patient brains showed reduced expression of γ, δ, c, and β subunit genes [123]. The subunits β, d, e, and F6 were down-regulated in the early-onset AD, as revealed by the iTRAQ quantitative mass spectrometric technique [124], whereas another proteomics analysis of hippocampal subcellular fractions from a murine AD model showed a decreased level of the peripheral stalk subunit d [125]. Interestingly, the d subunit gene was firstly thought to be genetic risk factor for AD in a genome wide association study [126]. 

On the contrary, increased expression was found for the ATP synthase α subunit gene in a Transgenic Swedish APP mouse (Tg2576) model for AD, with increased levels of amyloid plaque formation in the brain [127]. Moreover, a transgenic mice line (J20 Tg) producing a mutant form of APP, corresponding to the Swedish and Indiana familial forms of AD, showed a 12.2-fold increase in the α subunit level in a whole brain homogenate [128]. One might speculate that the increased subunit levels are due to an adaptive response only occurring in the AD animal models. Yet, in 2004 a study in AD patients by Manczak et al. showed increased mRNA levels for the mitochondrial *ATP6* and *ATP8* genes in brains, while increased levels of the δ subunit in the frontal cortex by immunofluorescence analysis [129]. 

ATP synthase is further controlled by possible interactions with other proteins modulating its activity and influencing neurodegeneration. Selective loss of the peripheral stalk subunit of ATP synthase, OSCP, was found in the brains of AD individuals and in an AD mouse model [130]. OSCP loss and complex interactions with Aβ leads to reduced ATP production, elevated oxidative stress and activated permeability transition [130]. The authors suggested that the restoration of OSCP ameliorates Aβ-mediated mouse and human neuronal mitochondrial impairments, including the effects on ATP synthesis and the resultant synaptic injury [130]. This finding demonstrating the OSCP involvement in AD is of particular interest, given that this subunit is the molecular-binding site for the CypD, the matrix prolyl-cis-trans-isomerase, which has been shown to modulate ATP synthase catalytic activity [91] and the ATP synthase transition to the permeability transition pore [131]. A previous study in an AD mouse model showed that neuronal and synaptic stress due to the interaction of CypD with mitochondrial Aβ are attenuated in CypD-deficient cortical mitochondria. CypD deficiency protected neurons from Aβ- and oxidative stress-induced cell death, in a mechanism involving the permeability transition pore [132], which was also confirmed in other AD transgenic mouse models [133]. Moreover, CypD levels, which increased in aging mice, have been shown to decrease ATP synthase activity and to promote mitochondrial dysfunction [134]. Compared with non-synaptic mitochondria, the synaptic mitochondria showed a greater degree of age-dependent accumulation of Aβ and deficits in mitochondrial function, as shown by increased mitochondrial permeability transition and decline in respiration [135]. In an AD animal model (5×FAD mice) the genetic depletion of CypD mitigates OSCP loss via ubiquitin-dependent OSCP degradation and attenuates OSCP/Aβ interaction preserving the ATP synthase function, mitochondrial bioenergetics and improved mouse cognition [136]. The authors’ interpretation is that CypD is a critical mediator that promotes OSCP deficits in AD-related conditions, providing a promising therapeutic strategy to correct mitochondrial dysfunction for AD therapy.

The most frequent form of neurodegenerative disorder affecting movement, PD, is caused by death of dopaminergic neurons in the mesencephalic region called substantia nigra pars compacta. In astrocytes derived from *PINK1*-knockout mice, proliferation defects were associated with a decrease in mitochondrial mass, membrane potential and ATP production as well as an increase in cellular ROS. Treatment of wild-type astrocytes with the ATP synthase inhibitor oligomycin was sufficient to mimic the proliferation phenotype observed in *PINK1*-deficient murine cells [137]. Protein aggregation and mitochondrial dysfunction are two central pathogenic processes in both familial and sporadic PD. However, the way in which these two processes converge to cause neurodegeneration was only recently proposed. Protein aggregation causes α-synuclein to switch from its physiological role to a pathological toxic gain of function form. Under physiological conditions, monomeric α-synuclein improves ATP synthase efficiency [138]. On the other hand, aggregation of α-synuclein monomers generates beta sheet-rich oligomers localized in the mitochondria in close proximity to several mitochondrial proteins including ATP synthase. Oligomers induce selective oxidation of the ATP synthase β subunit and mitochondrial lipid peroxidation. These oxidation events are proposed to increase the probability of permeability transition pore opening, triggering mitochondrial swelling and, ultimately, cell death [139]. Moreover, the protein DJ-1, linked to early onset PD, if defective, binds the ATP synthase β subunit. The interaction with the wild-type form of DJ-1 decreased the mitochondrial uncoupling and enhanced ATP production, while mutations in *PARK7* gene encoding DJ-1 (or *PARK7*-knockout) increased mitochondrial uncoupling and depolarized neuronal mitochondria [140]. The Authors suggested that this observation may depend on the presence of a leak at the level of the c-ring of ATP synthase in the membrane, which may be closed by pharmacological treatment [140].

ALS is an appalling neurodegenerative disease characterized by the loss of spinal motor neurons, which is rapidly progressive and lethal [141]. The most common genetic form of the disease is caused by GGGGCC repeat expansion in the *C9ORF72* gene. It was found that poly(GR) preferentially binds to the ATP synthase α subunit and promotes its degradation via the ubiquitin-proteasome pathway. Moreover, inducing the expression of *ATP5F1A* gene in poly(GR)-expressing neurons or reducing the poly(GR) level in adult mice after disease onset, rescued poly(GR)-induced neurotoxicity [142]. 

In a small subset of patients, the disease is caused by mutations in superoxide dismutase 1 [143]. Transgenic mice producing the mutant protein display mitochondrial alterations, including swelling, respiratory inhibition and an elevated generation of ROS [144,145]. Another form of ALS is caused by mutant forms of fused in sarcoma or translocated in liposarcoma (FUS), which is a multifunctional DNA/RNA-binding protein associated with neurodegeneration. In both cellular and animal models, the expression of wild-type or an ALS-associated mutant (p.Pro525Leu) FUS disrupts the formation of the mitochondrial ATP synthase supercomplexes and suppresses the activity of ATP synthase, resulting in mitochondrial cristae loss followed by mitochondrial fragmentation. Expression of FUS increases levels of the β subunit which is not properly assembled, and importantly, the downregulation of this subunit by RNA interference partially rescues neurodegenerative phenotypes [146]. In other studies, fibroblasts from patients affected by frontotemporal dementia and ALS presented mitochondrial ultrastructural alterations and fragmentation of the mitochondrial network together with respiratory chain deficiency. A missense mutation was identified (c.176C>T; p.Ser59Leu) in the *CHCHD10* gene which encodes a mitochondrial coiled-coil helix protein, whose function is unknown. Blue native-PAGE analysis of patient muscles revealed altered ATP synthase assembly which might contribute to the described abnormal organization of cristae morphology in these patients [147].

### 3.3. The c Subunit of ATP Synthase and Neurodevelopmental Disorders

Fragile X-related disorders are due to a dynamic mutation of the CGG repeat in the *FMR1* gene on chromosome X, encoding for the RNA-binding protein FMRP. In patients these disorders are associated to mental retardation and neurocognitive deficits. In primary human-derived fibroblasts, mitochondrial morphology is altered and displays “donut-shaped” organelles [148]. The mouse model of fragile X syndrome, the knockout of *FMR1*, resembled the human phenotype. Mouse brain mitochondria displayed a decreased ATP synthesis, while showing higher activities of the isolated respiratory chain complexes than in controls, suggesting a possible defect at the level of ATP synthase [149]. More recently, Elizabeth Jonas and coworkers showed that fragile X-affected neurons from mouse synthesized lower levels of cellular ATP [150]. These authors observed for the first time an increased level of the ATP synthase β subunit, an accumulation of the c subunit in insoluble aggregates in brain mitochondria and the presence of a proton leak in the F_o_ sector of the enzyme [150]. This finding can explain the decreased ATP synthesis in spite of a fully active respiratory chain shown in *FMR1-* knockout mouse [149]. The presence of c subunit aggregates was also revealed in the group of neuronal ceroid-lipofuscinoses diseases that are linked by common clinical and pathological features falling under the description of Batten disease. Although the ceroid-lipofuscinoses present pathologically as lysosomal storage diseases, there is severe but selective neurodegeneration that leads to the clinical signs of dementia, blindness, seizures, and premature death. It was the accumulation of subunit c of mitochondrial ATP synthase in lysosomes of ovine tissues that are models of ceroidlipofuscinosis that first drew attention to the possible association of mitochondrial dysfunction with the pathogenesis of these diseases. It was reasoned that accumulation of storage bodies containing the c subunit within lysosomes was the consequence of a defect in its catabolic pathway [151]. This hypothesis was extended to include its initial disassembly from the “F_o_ complex domain” of ATP synthase in the inner mitochondrial membrane [152]. This would be the initial step in the catabolic pathway and could depend on enzymes such as phospholipases rather than a protease [152]. Ca^2+^ causes a decrease of ATPase activity in isolated liver mitochondria from the ovine model of the disease, in comparison to controls displaying higher ATPase activity in the presence of Ca^2+^ [152], a fact that might be explained by proton leak but awaits further studies to be clarified. 

## 4. Conclusions

The mitochondrial ATP synthase is a multi-subunit complex fundamental for the mitochondrial function and ATP synthesis under physiological conditions. In this review, we gave an update on the involvement of this mitochondrial enzyme in human diseases, such as encephalo- and cardiomyopathies of mitochondrial or nuclear origin, cardiovascular, neurodegenerative diseases or neurocognitive disorders, ranging from those that are caused by specific ATP synthase gene mutations to those that are instead initiated by other factors but are promoted by dysfunctions in the enzyme assembly and catalytic activity. 

We here analyzed altered expressions of ATP synthase genes, enzyme subunit composition, post-translational modifications and interactions as causes of altered ATP synthase complex assembly and activity in human diseases, leading to mitochondrial morphology alterations and cell death.

## Figures and Tables

**Table 1:** Human pathogenic mutations occurring in ATP synthase subunits and assembly factors. The mutant subunits, or the mutant assembly factors of the ATP synthase found in human diseases are listed. Their specific nucleotide and amino acid substitutions and the related consequences on ATP synthase activity, assembly or mitochondrial morphology are summarized (nd, not defined).

ATP Synthase Subunit or Assembly Factor	mtDNA or nDNA Mutation	Protein Mutation	ATP Synthase			References
			***Activity***	***Assembly***	***Mitochondrial morphology***	
***ATP6*** **(a subunit)**	m.8993T>G	p.Leu156Arg	decreased	normal	nd	[29,30,31,32,33]
	m.8993T>C	p.Leu156Pro	decreased	nd	nd	[29,30,31]
	m.9176T>G	p.Leu217Arg	decreased	impaired	altered cristae	[28,34]
	m.9176T>C	p.Leu217Pro	decreased	impaired	altered cristae	[28,35]
	m.9035T>C	p.Leu170Pro	decreased	nd	nd	[36]
	m.9185T>C	p.Leu220Pro	decreased	nd	nd	[37,38,39]
	m.9191T>C	p.Leu222Pro	decreased	impaired (in the yeast model)	nd	[37,39]
	m.8969G>A	p.Ser148Asn	decreased	nd	nd	[40,41,42]
	m.8611_8612 insC	p.Leu29Profs*36	decreased	impaired	distorted mitochondria, aberrant cristae formation	[43]
***ATP6* (a subunit)** **and *ATP8*** **(A6L subunit)**	m.8528T>C	a p.Met1Thr + A6L p.Trp55Arg	decreased	impaired	nd	[44,45]
	m.8529G>A	a p.Met1Ile + A6L p.Trp55 *	decreased	impaired	nd	[46]
	m.8561C>G	a p.Pro12Arg + A6L p.Pro66Ala	decreased	impaired	nd	[47]
	m.8561C>T	a p.Pro12Leu + A6L p.Pro66Ser	decreased	impaired	nd	[48]
***ATP5F1E*** **(ε subunit)**	c.35A>G	p.Tyr12Cys	decreased	impaired	nd	[49]
***ATP5F1A*** **(α subunit)**	c.985C>T	p.Arg329Cys	decreased	impaired	nd	[50]
	c.962A>G	p.Tyr321Cys	decreased	nd	nd	[51]
***ATP5F1D*** **(δ subunit)**	c.245C>T	p.Pro82Leu	decreased	impaired	decreased number of cristae	[52]
	c.317T>G	p.Val106Gly	decreased	impaired	nd	[52]
***ATP5MK*** **(DAPIT subunit)**	c.87+1G>C	/	decreased	impaired	altered cristae shape	[53]
***ATPAF2***	c.280T>A	p.Trp94Arg	decreased	Impaired	normal	[54,55]
***TMEM70***	c.317–2A>G	/	decreased	impaired	different alterations including swollen, giant or small mitochondria; or irregularly shaped mitochondria (with concentric, fragmented or aggregated cristae)	[56,57,58,59]

Note. (*) indicates a STOP codon.
